# Age‐related variation in the trophic characteristics of a marsupial carnivore, the Tasmanian devil *Sarcophilus harrisii*

**DOI:** 10.1002/ece3.6513

**Published:** 2020-07-07

**Authors:** Olivia Bell, Menna E. Jones, Manuel Ruiz‐Aravena, Rodrigo K. Hamede, Stuart Bearhop, Robbie A. McDonald

**Affiliations:** ^1^ Environment and Sustainability Institute University of Exeter Penryn UK; ^2^ School of Natural Sciences University of Tasmania Hobart Tas. Australia; ^3^ Department of Microbiology and Immunology Montana State University Bozeman MT USA; ^4^ Centre for Ecology and Conservation University of Exeter Penryn UK

**Keywords:** isotopic niche, ontogeny, *Sarcophilus harrisii*, stable isotope analysis, Tasmanian devil, trophic niche

## Abstract

Age‐related changes in diet have implications for competitive interactions and for predator–prey dynamics, affecting individuals and groups at different life stages. To quantify patterns of variation and ontogenetic change in the diets of Tasmanian devils *Sarcophilus harrisii*, a threatened marsupial carnivore, we analyzed variation in the stable isotope composition of whisker tissue samples taken from 91 individual devils from Wilmot, Tasmania from December 2014 to February 2017. Both δ^13^C and δ^15^N decreased with increasing age in weaned Tasmanian devils, indicating that as they age devils rely less on small mammals and birds, and more on large herbivores. Devils <12 months old had broader group isotopic niches, as estimated by Bayesian standard ellipses (SEA_B_ mode = 1.042) than devils from 12 to 23 months old (mode = 0.541) and devils ≥24 months old (mode = 0.532). Devils <24 months old had broader individual isotopic niches (SEA_B_ mode range 0.492–1.083) than devils ≥24 months old (mode range 0.092–0.240). A decrease in δ^15^N from the older whisker sections to the more recently grown sections in devils <24 months old likely reflects the period of weaning in this species, as this pattern was not observed in devils ≥24 months old. Our data reveal changes in the isotopic composition of devil whiskers with increasing age, accompanied by a reduction in isotopic variation both among population age classes and within individuals, reflecting the effect of weaning in early life, and a likely shift from an initially diverse diet of small mammals, birds, and invertebrates towards increasing consumption of larger herbivores in adulthood.

## Graphical abstract

By using stable isotope analysis of whiskers, we quantify variation in the isotopic trophic niches of Tasmanian devils as they age. We highlight changes in the isotopic composition and variation of devil whiskers with increasing age, reflecting the effect of weaning in early life, and a likely shift from a diverse subadult diet of small mammals, birds and invertebrates through to larger herbivores in adulthood.

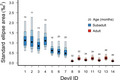

## 1 INTRODUCTION

Intraspecific variation in dietary niches can relate to age, sex, or morphological classes, or result from individual specialization (Bolnick et al., 2003; Polis, 1984), with consequences for individuals, populations, and communities. Such dietary niche variation can affect individual fitness, through differences in breeding behavior and reproductive outcomes (Anderson et al., 2009) or differential exposure to pathogens, parasites, and predators (Johnson et al., 2009). Intraspecific dietary variation also has importance for conservation if individuals, sex, or age classes are differentially exposed to threats, for example, use of fisheries discards and entanglement in seabirds (Votier et al., 2010), or in cases where management actions selectively target groups or individuals that have larger impacts upon human interests, for example, by identifying and managing “problem individuals” (Swan, Redpath, Bearhop, & McDonald, 2017).

As organisms age, they might experience changes in physiological constraints and competitive pressures, resulting in different realized niches and/or diets at different life stages (Polis, 1984; Werner & Gilliam, 1984). Ontogenetic dietary niche shifts have been well documented in taxa that undergo major morphological, physiological, or behavioral shifts during maturation, such as invertebrates, fish, and reptiles (Olson, 1996; Reich, Bjorndal, & Bolten, 2007; Werner & Gilliam, 1984). Mammals experience less extreme ontogenetic changes, yet associated developmental differences in diets will have ramifications for the intensity of intra‐ and interspecific interactions at different life stages. Clearly, mammals undergo dietary change during infancy, when they are weaned from a diet of maternal milk to solid foods (Geipel, Kalko, Wallmeyer, & Knörnschild, 2013; Lee, Majluf, & Gordon, 1991). Even after this point, body size and experience may place additional constraints on diets, particularly for mammalian predators that must find, catch, subdue, and consume their prey. Studies of dietary niche development and weaning in mammals have largely focussed on eutherian mammals, both marine (Knoff, Hohn, & Macko, 2008; Newsome, Etnier, Monson, & Fogel, 2009; Orr, Newsome, Laake, Vanblaricom, & Delong, 2012; Riccialdelli et al., 2013) and terrestrial (Fahy et al., 2014; Geipel et al., 2013). In contrast, relatively little is known about within‐species age‐related variation in dietary niches in marsupials (Albanese, Dacar, & Ojeda, 2012; Martins, Araújo, Bonato, & dos Reis 2008).

**FIGURE 1:**
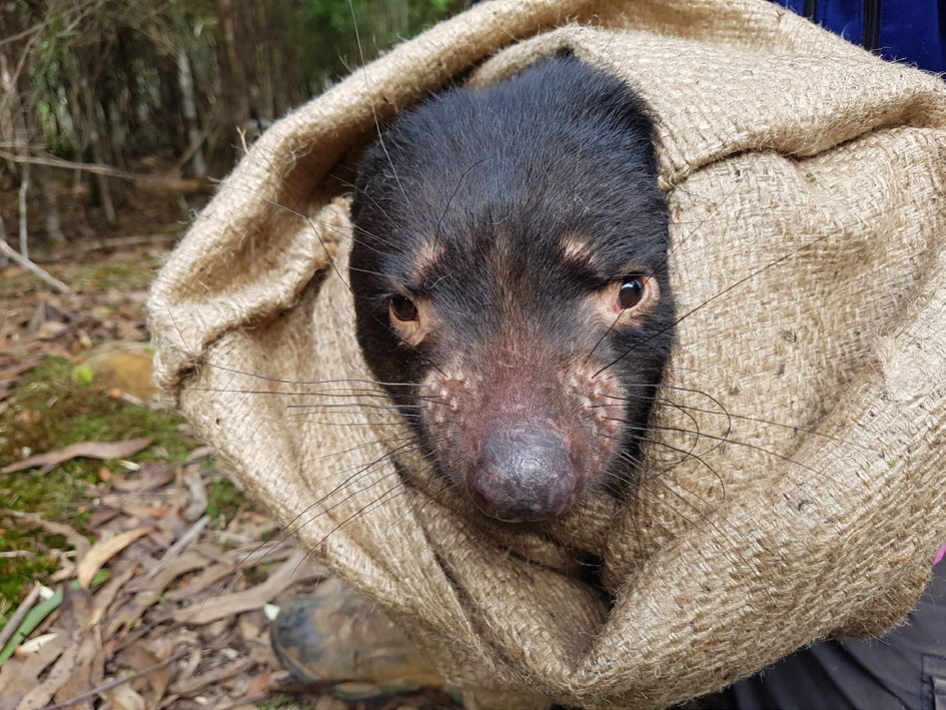
Tasmanian devil *Sarcophilus harrisii* showing the large vibrissae (whiskers) used in stable isotope analysis of variation in isotopic niche. Photograph Olivia Bell

Tasmanian devils *Sarcophilus harrisii* (Figure 1) are the largest extant carnivorous marsupial (Order Daysuromorphia) and are now restricted to the Australian island state of Tasmania. As the largest terrestrial mammalian predator in Tasmanian ecosystems, Tasmanian devils (hereafter, “devils”) exert top‐down influences on the behavior and abundance of sympatric mesopredators and herbivores (Cunningham et al., 2018; Cunningham, Johnson, Hollings, Kreger, & Jones, 2019; Hollings, Jones, Mooney, & Mccallum, 2014; Hollings, McCallum, Kreger, Mooney, & Jones, 2015). Since 1996, populations of Tasmanian devils have experienced severe decline associated with the emergence of devil facial tumor disease (DFTD), a transmissible cancer that is fatal in most cases (Loh et al., 2006; Pearse & Swift, 2006; Pye et al., 2016; Pyecroft et al., 2007). DFTD is characterized by tumors around the face, neck, and mouth (Hawkins et al., 2006). Transmission occurs through injurious biting interactions, mostly during the mating season (Hamilton et al., 2019), with mortality of most infected adults by 3 years of age (Lachish, Jones, & McCallum, 2007; McCallum et al., 2009). Increased juvenile growth rates in response to reduced density in affected populations mean that about 50% of female devils are able to reach sexual maturity in their first year of independent life, instead of at 2 years old (Jones et al., 2008; Lachish et al., 2007; Lachish, McCallum, & Jones, 2009). The location of DFTD tumors on the mouth and face means that starvation can be a cause of death in DFTD‐infected devils, alongside metastasis‐related organ failure and secondary infections (Loh et al., 2006). Through disruption to normal feeding ability and metabolic demand as tumors grow in size, DFTD progression may cause changes in the diets of infected devils, with eventual implications for predator–prey and competitive interactions. Furthermore, individual dietary specialization in healthy individuals might translate into differences in susceptibility to DFTD, if different diets carry different risks of competitive encounters and potential disease transmission. Information on intraspecific dietary variation in devils is therefore desirable to consider potential interactions between DFTD, devil population decline, feeding, and community ecology.

Tasmanian devils are facultative scavengers. Although they are usually solitary, they often feed communally on larger carcasses, making such episodic feeding an important arena of conspecific interaction, competition, and, potentially, disease transmission. Scat content analyses suggest devils primarily eat the macropodid herbivores: Bennett's wallabies *Macropus rufogriseus* (average mass of males = 20 kg, females = 14 kg) and Tasmanian pademelons *Thylogale billardierii* (males = 7 kg, females = 4 kg) (Andersen, Johnson, Barmuta, & Jones, 2017; Jones & Barmuta, 1998; Strahan, 1983). Jones and Barmuta (1998) identified dietary differences between juvenile, subadult, and adult devils that were associated with age‐related body size and climbing ability (Jones & Barmuta, 2000); younger devils ate more birds and small mammals (categorized as antechinus *Antechinus* spp. to sugar glider *Petaurus b. breviceps ~ *35–140 g) and fewer large mammals (Bennett's wallaby to bare‐nosed wombat *Vombatus ursinus*, ~14–35 kg). Seasonal differences in diet were also found, with devils, particularly males, eating larger prey items in summer compared to winter (Jones & Barmuta, 1998). This is the extent of knowledge of intraspecific dietary variation in devils, and to our knowledge, no study has investigated dietary variation at the individual level.

Ontogenetic change in devil diets could be driven by behavioral or morphological development. As devils mature, they encounter multiple food types and gain foraging experience, which could increase foraging efficiency and selectiveness. For example, adult European hedgehogs *Erinaceus europaeus* exhibit narrower dietary niches than juveniles and tend to feed on larger prey, likely due to greater experience and more efficient foraging (Dickman, 1988). A relaxation of anatomical constraints in body size and morphology could also cause devil dietary niche characteristics to change with age. Tasmanian devils have one of the strongest bite forces (relative to body mass) of any extant predator, with strong canines and musculature enabling the delivery of crushing bites and consumption of bone (Attard, Chamoli, Ferrara, Rogers, & Wroe, 2011; Jones, 1997; Wroe, McHenry, & Thomason, 2005). Another specialized mammalian scavenger, the spotted hyaena *Crocuta crocuta*, does not achieve full adult skull development until after sexual maturity, due to the strong skull morphology and musculature required for cracking through bone, crucially restricting feeding speed in juvenile and young hyaenas (Tanner, Zelditch, Lundrigan, & Holekamp, 2010). Younger Tasmanian devils may be less capable of killing and/or consuming larger prey species, placing younger devils at a disadvantage, as large carcasses can be a focus for intraspecific competition (Hamede, McCallum, & Jones, 2008; Jones, 1995; Pemberton & Renouf, 1993). Therefore, both behavioral and anatomical disadvantages may lead young devils to incorporate different, smaller, or more diverse prey types into their diets, when compared to adults.

Stable isotope analysis has been widely applied to describe intraspecific variation in resource use, including ontogenetic dietary changes (Hammerschlag‐Peyer, Yeager, Araújo, & Layman, 2011; Inger et al., 2006; Newsome, Etnier, et al., 2009; Vales, Cardona, García, Zenteno, & Crespo, 2015), sex‐related differences in resource use (Phillips, McGill, Dawson, & Bearhop, 2011; Stauss et al., 2012), and individual specialization (Bodey et al., 2018; Newsome, Tinker, et al., 2009; Patrick et al., 2015; Robertson, McDonald, Delahay, Kelly, & Bearhop, 2014). Ratios of stable isotopes, δ^13^C and δ^15^N, in consumer tissues reflect those of their food resources, albeit with alteration following physiological processes associated with digesting, assimilating and routing these resources (Bearhop, Waldron, Votier, & Furness, 2002; Crawford, McDonald, & Bearhop, 2008; DeNiro & Epstein, 1978; Hobson & Clark, 1992). In broad terms, carbon isotopic signatures (δ^13^C) are influenced by the photosynthetic pathway of resources at the base of the food web, alongside environmental variables, while nitrogen signatures (δ^15^N) become enriched through the food web, reflecting an organism's trophic level. For example, weaning juvenile mammals are expected to exhibit higher δ^15^N than older individuals, as suckled young are feeding on milk derived from maternal tissues and so their apparent trophic level (inferred from stable isotope analyses) should reduce upon weaning (Evacitas, Kao, Worthy, & Chou, 2017; Hobson & Sease, 1998; Newsome, Tinker, et al., 2009; Orr et al., 2012). Isotopic niches of individuals and groups can be used as a proxy for elements of the ecological niche (Bearhop, Adams, Waldron, Fuller, & Macleod, 2004; Layman et al., 2012; Newsome, Martinez del Rio, Bearhop, & Phillips, 2007; Sheppard et al., 2018), as stable isotopes reflect the resources consumed and habitats used to obtain resources (bionomic and scenopoetic niche axes, respectively) and variability in these both within and among individuals (Bolnick et al., 2003). Furthermore, a time series of information for individuals can be generated by repeat sampling of tissues or, less invasively, by subsampling along the length of inert tissues, such as whiskers, that grow continuously and so reflect diet at the time of their synthesis. Importantly, stable isotope analysis also reflects assimilated diet, thereby incorporating easily digestible food sources that might be missed, or differently represented, by stomach and scat contents analysis of undigested or hard parts. In the case of Tasmanian devils, where multiple scat content analyses have reached similar conclusions regarding the primary diet of devils across multiple years and study sites (Andersen et al., 2017; Jones & Barmuta, 1998), stable isotope analysis enables further insight into the dietary niche characteristics of devil populations and individuals.

Using whisker samples collected from adult and subadult Tasmanian devils at a site affected by DFTD, we applied stable isotope analysis to quantify variation in the isotopic trophic niches of Tasmanian devils in three ways: (a) among‐individual variation, (b) population niche structure, and (c) within‐individual variation, in each case considering potential drivers of such variation, including age.

## 2 METHODS

### 2.1 Sample collection

Ninety‐one Tasmanian devils were sampled during trapping surveys undertaken every 3 months between December 2014 and February 2017 at Wilmot (41°23′07.8″S 146°10′02.9″E) in north‐west Tasmania. The Wilmot field site is 39.92 km^2^ in area and is composed of 28% native eucalypt forest, 42% eucalypt plantation, 28% agricultural land, and 2% other vegetation.

One whisker was collected from each devil by cutting close to the skin with scissors. Devils were individually identified by insertion of microchip transponders. Sex and weight were recorded. DFTD status was recorded based on visual diagnosis of clinical signs (tumors on the face, neck, or in the oral cavity) (Hawkins et al., 2006). Devils were assigned a year of birth using canine eruption, molar eruption, and tooth wear, which is accurate until the animal reaches 3 years of age (M.E. Jones, unpublished data). Tasmanian devils mate in late February/early March (Hesterman, Jones, & Schwarzenberger, 2008), with birth following several weeks later; therefore, all devils were given a standardized birth date of 1st April so that age in months could be approximated.

### 2.2 Whisker growth rates

Stable isotope analysis of whiskers involves subsampling whiskers into sections. To estimate the approximate time interval represented by a single whisker and the subsections, we measured whisker growth rates in a feeding trial with five captive Tasmanian devils (of a range of ages from 1.5 to 7 years old), held at Bonorong Wildlife Sanctuary, Tasmania. We fed the devils baits laced with Rhodamine B, which is a biomarker that is integrated into keratinous tissue after ingestion, leaving a distinctive band that can be viewed under a fluorescence microscope (Fisher, 1999). Devils were given a RhB dose of 50 mg/kg body weight, in a gelatine capsule hidden inside a dead day‐old chick. After a minimum of 2 weeks, two whiskers per devil were removed under general anesthetic during routine veterinary assessments. One devil underwent this procedure three times over a 9 month period, two twice and two only once. Therefore, a total of 18 whiskers from five devils sampled over three occasions were examined under a fluorescence microscope, and growth rate (in mm per day) was calculated by measuring the distance between the base of the whisker and the basal edge of the fluorescent band, dividing this by the number of days between bait consumption and whisker sampling.

### 2.3 Laboratory procedures

Whiskers collected for isotope analysis were rinsed with distilled water, air‐dried, and placed in a freeze dryer for 24 hr. Chopped whisker pieces with total mass of 0.7 ± 0.1 mg were weighed into tin cups for analysis. To investigate among‐individual variation and population niche structure, the basal section of each whisker was used as this likely represents the season in which devils were captured. To investigate within‐individual variation, the whiskers of 14 individuals were sampled in their entirety, resulting in between 9 and 15 subsamples per whisker, each representing periods further back in time, moving from the recently grown whisker base to the older whisker tip. Isotope analyses were conducted using a Sercon Integra‐2 elemental analyser isotope‐ratio mass spectrometer at the University of Exeter, and Thermoquest EA1110 elemental analyser linked to a Europa Scientific 2020 isotope‐ratio mass spectrometer at Elemtex Ltd, Cornwall UK. In both instances, samples were scale corrected using USGS40 and USGS41 with additional internal standards of bovine liver (University of Exeter and Elemtex Ltd) and alanine (University of Exeter only). Averaging across standards and laboratories, precision was 0.11‰ ± 0.02 (1 standard deviation ± standard error) and 0.13‰ ± 0.02 for δ^13^C and δ^15^N, respectively.

### 2.4 Statistical analysis

All analyses were conducted in R Version 3.5.2 (R Core Team, 2018).

### 2.5 Among‐individual variation

To test the correlates of isotopic variation among Tasmanian devils, linear models were fitted with δ^13^C and δ^15^N as response variables. Models included the fixed effects: age (in months), sex, DFTD infection status (binary), season and year, as well as interactions between season and year, age and sex, age and DFTD infection, and sex and DFTD infection. Season was categorized as summer (1st October to 31st March) or winter (1st April to 30th September). Year was fitted as a factor with each year (1, 2, 3) beginning on the 1st October, to reflect yearly trapping cycles starting from October 2014 onwards. Linear models were fitted with Gaussian error structure and identity link, and top model sets were generated using the R package MuMIn (Bartoń, 2018) and selected on the basis of increases in the Akaike information criterion corrected for sample size (AIC_c_), where ΔAIC < 2.

### 2.6 Population niche structure

As our linear models revealed the importance of age in influencing variation in both δ^13^C and δ^15^N, the population was divided into three age classes: age class 1 (subadults <12 months, *n* = 21), class 2 (subadults of 12–23 months, *n* = 31), and class 3 (adult devils ≥24 months, *n* = 39). Subadult devils were divided into these two age classes, as subadults under 12 months are certainly immature, whereas occurrences of precocial breeding can occur from 12 months in populations with high prevalence of DFTD (Jones et al., 2008; Lachish et al., 2009). Age class 1 included only two devils (both 8 months old) that would not have been weaned and independent of their mother upon capture. To estimate isotopic trophic niche areas for each age class, Bayesian standard ellipses (SEA_B_) were fitted around bivariate δ^13^C and δ^15^N data from basal whisker sections using the R package SIBER (Jackson, Inger, Parnell, & Bearhop, 2011). Calculations of SEA_B_ provide an estimate of uncertainty, with greater uncertainty where sample size is small. We also calculated standard ellipses corrected for sample size (SEA_C_); this niche metric captures similar niche properties as SEA_B_, but is better suited to graphical depiction of niche variation. Dispersal is male‐biased in Tasmanian devils, and genetic evidence indicates female dispersal has reduced in frequency or distance in areas affected by DFTD (Lachish, Miller, Storfer, Goldizen, & Jones, 2011), so Bayesian standard ellipses (SEA_B_) were also fitted for the two sexes separately in each age class to assess whether sex and dispersal relates to any differences in niche characteristics between age classes.

### 2.7 Within‐individual variation

To examine within‐individual variation, we fitted Bayesian standard ellipses (SEA_B_) for 14 individual devils, using bivariate δ^13^C and δ^15^N datapoints generated by sampling all sections of the individuals' whiskers. For this analysis, individuals were divided into two age classes: “subadult” (<24 months, *n* = 7) and “adult” (≥24 months, *n* = 7). Two, rather than three, age classes were used for this analysis due to small sample size. Broad isotopic niches in this instance could suggest a generalized diet through the period reflected by our data, or could reflect gradual shifts in diet over time. To examine how the isotopic composition of individual whiskers varied through time, we sequentially plotted the mean of the two most basal whisker sections, the two mid whisker sections, and the final two sections at the tip of the whisker.

## 3 RESULTS

### 3.1 Whisker growth rates

Of 18 whiskers from 5 individual Tasmanian devils, 13 displayed a fluorescent band, with an average growth rate of 0.48 mm/day (range 0.27–0.69 mm/day). The remaining 5 whiskers showed fluorescence at the very base of the whisker, suggesting whiskers are retained for a period of time after growth is complete. As fewer than one third of the whiskers were retained, this is likely to occur for a relatively short period of time. Our sample size was too small to test any potential relationship between age and whisker growth rate. Applying this growth data to the 14 whiskers fully subsampled to analyze within‐individual variation, our data represent between 148 and 264 days of growth (mean 194 days), or approximately 5–9 months of diet, with each subsection representing a median of 10 days growth (95% quantiles = 6–46 days). Applying this growth rate to basal whisker sections sampled to investigate among‐individual variation and population niche structure, the median time represented by each section is 6 days (95% quantiles = 4–13 days). Assuming a constant rate of growth, subsections of 14 whiskers fully sampled to investigate within‐individual variation represent a greater median number of days comparative to the basal sections sampled to investigate among‐individual variation and population niche structure, as whiskers become thinner from the base to the tip; therefore, subsections also become longer.

### 3.2 Among‐individual variation

**FIGURE 2:**
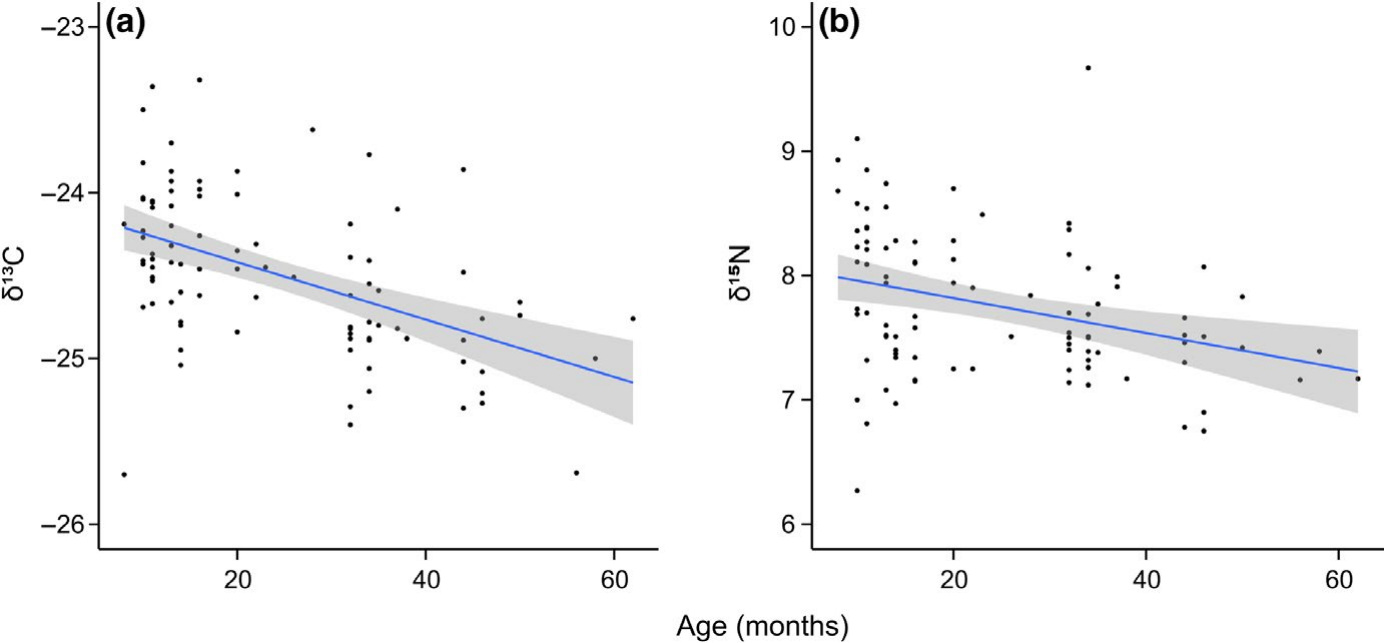
Relationships between Tasmanian devil age and (a) δ^13^C and (b) δ^15^N values of whisker samples from 91 individuals. The plotted lines represent linear regressions with standard error

For 91 individuals, δ^13^C values ranged from −25.7‰ to −23.3‰ (mean −24.5‰), while δ^15^N values ranged from 6.3‰ to 9.7‰ (mean 7.8‰). Age and year were important correlates of variation among devils in both δ^13^C and δ^15^N (*n* = 90 after removal of missing values) (Table 1). Only one model, which included age and year, featured in the top model set for δ^13^C. Age and year were retained in all four top models of variation in δ^15^N (relative variable importance = 1 for both variables), with season retained in three (RVI = 0.79), and sex (RVI = 0.20) and an interaction between the effects of season and year (RVI = 0.17) each featuring in only one model. Both δ^13^C and δ^15^N decreased with increasing age (Figure 2). As devil reproductive ecology means the youngest devils (<12 months) could only have been trapped in summer, we checked for correlation between age and season; however, effects were consistent between single term models and models with both terms included (Table 1). δ^15^N steadily increased through the three study years, and δ^13^C increased in year 2 compared to year 1, with year 3 sitting between the two earlier years.

**TABLE 1:** Summary of analyses of variation in stable isotope ratios of Tasmanian devil whiskers from Wilmot, Tasmania

Response	Model rank	Intercept	Year		Age	Season	Sex	Season: Year		DFTD	*df*	logLik	ΔAICc	Weight
			Year 2	Year 3				Season: Year 2	Season: Year 3					
δ^13^C	1	−24.63	0.47	0.13	−0.41						5	−39.11	0	1
δ^15^N	1	7.61	0.43	0.25	−0.36	−0.25					6	−69.29	0	0.36
	2	7.62	0.36	0.33	−0.32						5	−71.10	1.33	0.18
	3	7.61	0.43	0.25	−0.34	−0.24	0.11				7	−68.81	1.40	0.18
	4	7.62	0.47	0.26	−0.36	−0.18		−0.21			7	−68.98	1.73	0.15
	RVI		1	1	1	0.79	0.20	0.17						

Note

Basal sections of whiskers from 90 Tasmanian devils were analyzed for δ^13^C and δ^15^N. The table summarizes the top model set for each general linear model of variation in δ^13^C and δ^15^N, where top models sets were restricted to models with ΔAICc < 2. Effect sizes are reported for retained variables in each model. Relative variable importance (RVI) is shown for δ^15^N as multiple models had ΔAICc < 2.

### 3.3 Population niche structure

**FIGURE 3:**
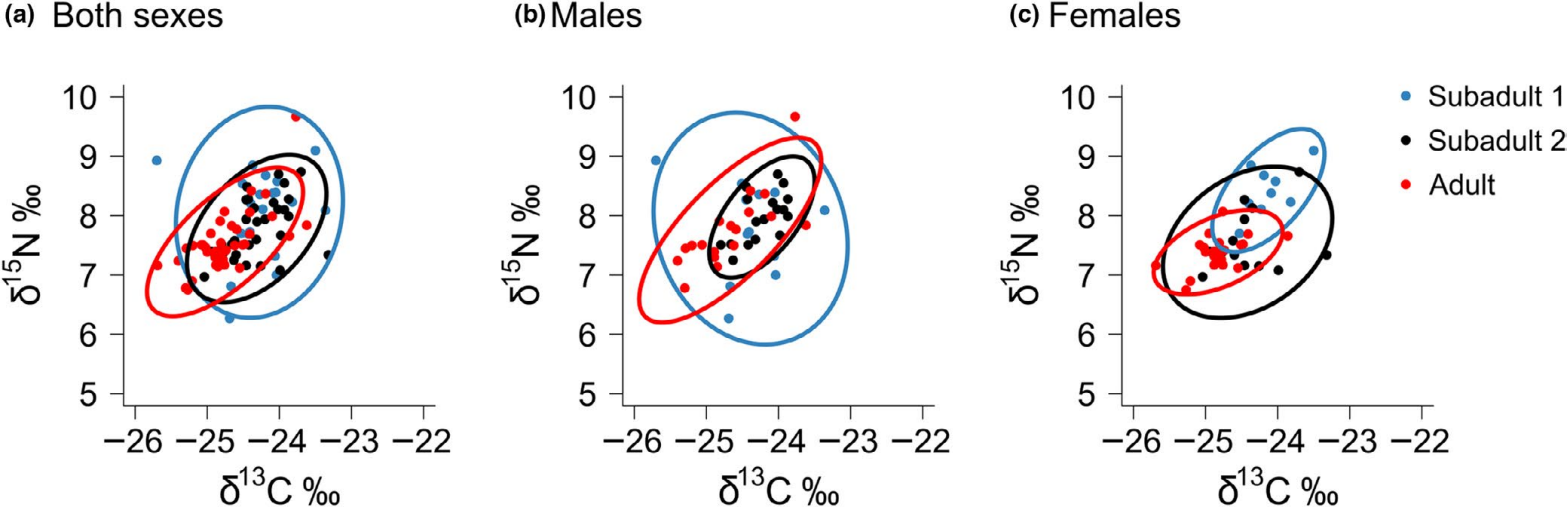
Values of δ^13^C and δ^15^N from analysis of Tasmanian devil whisker samples and isotopic niches by sex and age class. (a) Male and Female devils, (b) Male devils and (c) Female devils. Three age classes are subadult age class 1 (<12 months; blue), subadult age class 2 (12–23 months; black) and adults (≥24 months; red). Isotopic niches are standard ellipses corrected for sample size (SEA_C_)

Devils in age class 1 (<12 months) had a greater isotopic niche area, as a group (SEA_B_ mode = 1.042, 95% CI = 0.664–1.627, SEA_C_ = 1.130) compared to those in age class 2 (12–23 months; SEA_B_ mode 0.541, 95% CI = 0.383–0.794, SEA_C_ = 0.576) and adults in age class 3 (≥24 months; SEA_B_ mode 0.532, 95% CI = 0.381–0.727, SEA_C_ = 0.541) (Figure 3). Male devils in age class 1 had a greater isotopic niche area as a group than female devils (male SEA_B_ mode = 1.288, 95% CI = 0.697–2.385; female SEA_B_ mode = 0.325, 95% CI = 0.161–0.716). Similarly, male devils in age class 3 (adults) had a greater isotopic niche area than females (male SEA_B_ mode = 0.701, 95% CI = 0.433–1.143; female SEA_B_ mode = 0.291, 95% CI = 0.184–0.447). However, in age class 2, females had a greater isotopic niche area than males (male SEA_B_ mode = 0.312, 95% CI = 0.189–0.516; female SEA_B_ mode = 0.696, 95% CI = 0.415–1.293).

### 3.4 Within‐individual variation

**FIGURE 4:**
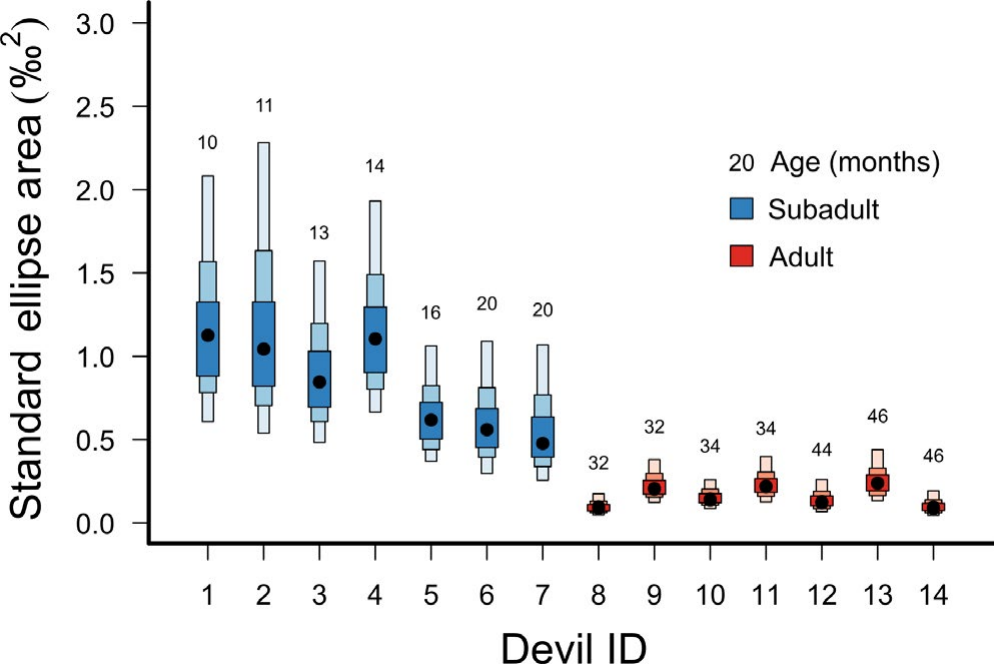
Summary of isotopic niche areas for 14 individual subadult and adult Tasmanian devils. 7 subadults were <24 months old and 7 adults were >24 months. Individuals are shown in order of increasing age from left to right. Age (in months) is indicated above each individual standard ellipse area estimation. Isotopic niche areas are Bayesian standard ellipse area (SEA_B_) estimates. SEA_B_ mode estimates are represented by black dots, while inner, middle and outer boxes represent 50%, 95% and 99% credible intervals, respectively

**FIGURE 5:**
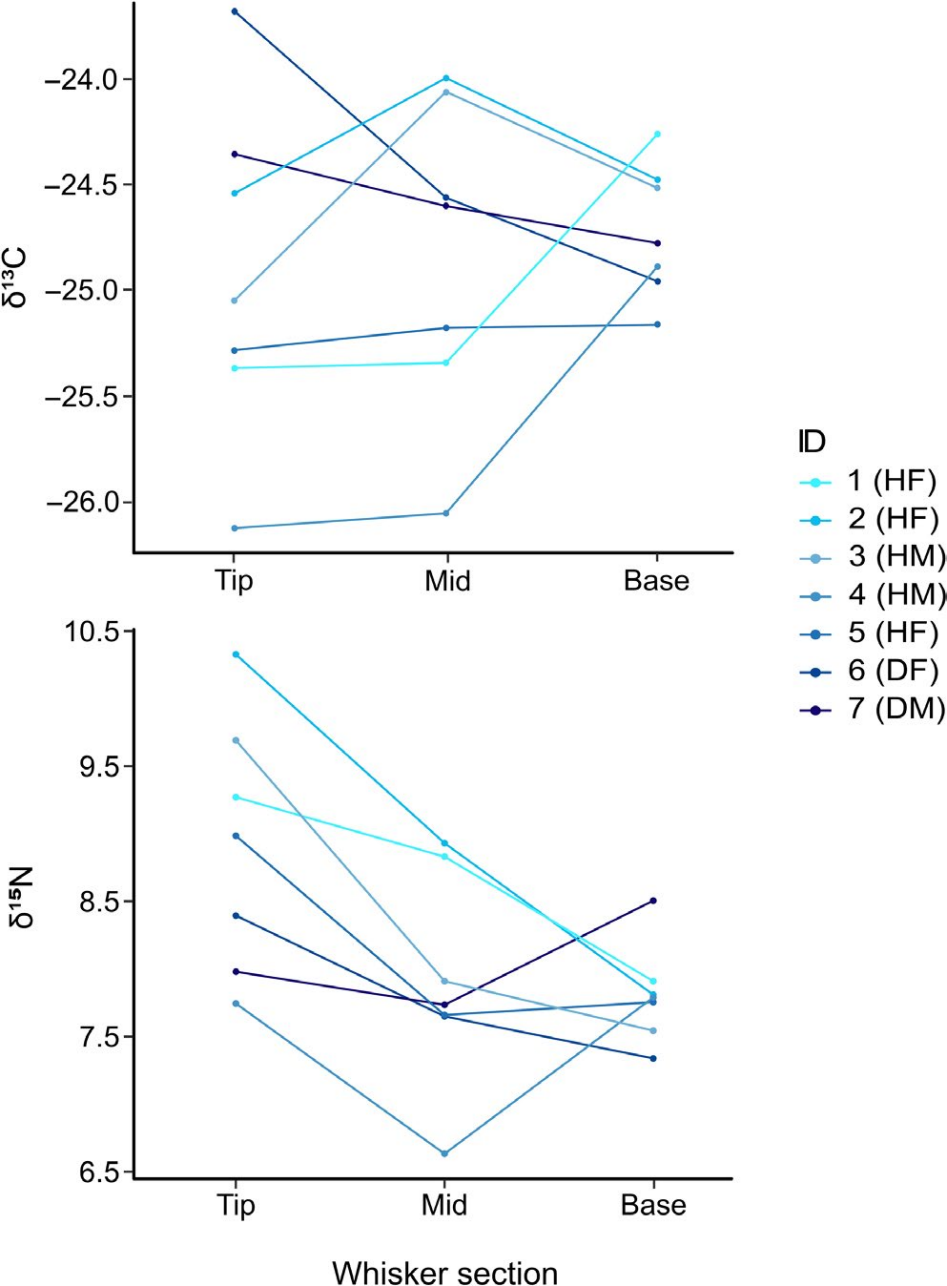
Summary of δ^13^C and δ^15^N values from the tip to the base of whiskers from seven subadult Tasmanian devils. Subadult devils in this analysis are <24 months old. The tip is the distal and oldest section of the whisker while the base is the most recently grown. Devil ID corresponds with those in Figure 4 and incorporates disease (D = DFTD diagnosed, H = DFTD‐free) and sex (F = female, M = male)

Subadult devils (<24 months) exhibited greater within‐individual isotopic niche areas (SEA_B_ mode range 0.492–1.083) than adult devils (≥24 months) (SEA_B_ mode range 0.092–0.240), with individual isotopic niche areas (SEA_B_) decreasing with age over the period 10–20 months (Figure 4). This greater variability in subadult isotopic niches was also apparent along the axes of both δ^13^C (average range of subadults 1.51‰ and adults 0.68‰) and δ^15^N (average range of subadults 2.23‰ and adults 1.01‰). Variation in δ^13^C and δ^15^N between subadult individuals declined over time, with a reduction in variation between individuals from the distal end (tip) to the base of the whiskers (Figure 5); in δ^15^N, this was accompanied by a reduction in mean δ^15^N from 9.13‰ to 7.90‰. By contrast, mean δ^15^N among adult devils stayed relatively constant over time, changing from 7.45‰ at the distal end (tip) to 7.35‰ at the base of the whiskers.

## 4 DISCUSSION

Our results reveal Tasmanian devils exhibit ontogenetic changes in isotopic niche characteristics. Among‐individual variation was largely driven by a decrease in δ^13^C and δ^15^N values with advancing age. Population niche structure and within‐individual variation also changed with age, as isotopic niche areas of population age classes and of individuals both decreased with increasing age. Using isotopic niche as a proxy for dietary niche, we interpret this as indicating broader dietary niches in subadult devils both as an age class and as an individuals.

Decreasing isotopic niche areas of devils from early independence (subadult age class 1) through to adulthood was driven by a contraction toward the lower end of δ^15^N values, rather than a shift of a consistently broad niche toward lower trophic levels. Maternal milk, with elevated δ^15^N values, will likely have contributed to the higher δ^15^N values of the youngest devils. Alongside any effects of weaning, higher δ^15^N in younger Tasmanian devils and the age‐related decline could also reflect diets in younger animals that are higher in trophic level, incorporating medium to small mammals and birds, with larger, herbivorous mammals increasing in their importance in diets as the devils age. An ontogenetic shift in diet as devils grow from subadult to adult body size would also contribute to the observed contraction in isotopic trophic niches. Of the prey base available to Tasmanian devils, the larger marsupials are herbivorous, including the bare‐nosed wombat, and the macropodid Bennett's wallaby and Tasmanian pademelon, and the smaller mammals are omnivores to varying degrees, including the potoroid macropods (long‐nosed potoroo *Potorous tridactylus apicalis* and eastern bettong *Bettongia gaimardi*) and peramelids or bandicoots (eastern barred bandicoot *Perameles gunnii* and southern brown bandicoot *Isoodon obesulus*) (Bennett & Baxter, 1989; Quin, 1988; Taylor, 2006). A high proportion of bird species in Tasmania are also insectivorous (Ridpath & Moreau, 1965). Jones and Barmuta (1998) found the diet of subadult devils, based on scat contents, contained a lower biomass of large mammal prey species and a higher biomass of birds, relative to adult devils which fed primarily on larger mammals. Unweaned juvenile devils (defined in Jones and Barmuta (1998) as first year young trapped between October and January that were still suckling) were also found to have a higher percentage of small mammals and birds in their diet than adult devils. The observed differences in isotopic niche size and position through ontogeny in Tasmanian devils could therefore be the result of changes in the type of food resources consumed, as well as the effect of weaning.

Isotopic signatures in consumer tissues can reflect spatial or temporal variation in the environmental isotopic baseline, rather than actual variation in resources consumed. If ignored, this can present a problem for the interpretation of isotopic data (Cummings, Buhl, Lee, Simpson, & Holmes, 2012). However, with application of prior ecological knowledge, variation in the isotopic baseline can be an advantage, for example in investigating the consistency of foraging habitat use in the face of ecological change (Bodey, Bearhop, Roy, Newton, & McDonald, 2010). Upon reaching independence, Tasmanian devils disperse from the natal den. Dispersal in devils is male‐biased, similar to other mammalian species, although both sexes disperse (Lachish et al., 2011). As such, greater isotopic heterogeneity in subadults could be driven by mobile, newly independent devils passing through our study site, having foraged in multiple locations outside of the immediate study area. A reduction in female dispersal has been found in sites where DFTD is present, although this has not affected male dispersal (Lachish et al., 2011). Subadult males (age class 1) did exhibit broader isotopic niches than females. On the other hand, genetic and observational data suggest the majority of devil dispersal events occur over distances of 14–30 km (Lachish et al., 2011). Our study site, Wilmot (almost 40 km^2^), is situated within a relatively uniform landscape of native and plantation forest and agriculture, reducing the likelihood of large changes in isotopic baseline within the trapping area, although some localized environmental heterogeneity may still occur.

Subadult devils had a broader individual isotopic niche than adults, along both the δ^15^N and the δ^13^C axes. The depletion in δ^15^N from the older whisker sections to the base of the whisker in young Tasmanian devils (Figure 5) is evidence of the weaning process in Tasmanian devils. Tasmanian devils are born after approximately 3 weeks gestation (Keeley et al., 2017), when the young crawl into the mother's pouch and attach to one of four available teats. Juveniles remain in the pouch until they are 5–6 months old, at which point they are left in the den (Guiler, 1970). Records of lactating adult females suggest weaning is completed approximately 10 months after birth, coinciding with independence and dispersal from the natal den (Pemberton, 1990). The process of weaning has been predicted to result in an enrichment of δ^13^C, on account of the high lipid content in milk; lipids are depleted in δ^13^C relative to proteins and carbohydrates (Tieszen, Boutton, Tesdahl, & Slade, 1983). However, the evidence for this in eutherian mammals is mixed (Knoff et al., 2008; Newsome, Etnier, et al., 2009; Orr et al., 2012; Vales et al., 2015). Furthermore, this prediction may not hold for marsupial species. Eutherian mammals have a long gestation, and neonates are precocial in comparison with marsupial neonates, which are born before the completion of organogenesis. To match the evolving needs of a suckling marsupial juvenile, milk composition in marsupials changes through lactation; for example, lipid and protein content increases while carbohydrate content decreases in late stage lactation in the herbivorous tammar wallaby *Macropus eugenii* (Pharo, 2019). That said, as devils in our analysis of within‐individual variation were at least 10 months old, whiskers are most likely to represent the second stage of lactation, where devil joeys are capable of life outside of the pouch, at which point milk composition may be more stable and similar to eutherian milk. A more likely explanation for δ^13^C variation in individual diets of young devils would be that an increasing variety of solid food resources was being consumed as weaning progressed. Prior to independence, young individuals roam increasing distances from the natal den, gradually increasing their dietary independence and mothers also bring some food back to the den (M.E. Jones, unpublished trapping records at and near dens).

Devil Facial Tumour Disease infection status was not included in any of our top models (Table 1). It may be that our binary measure of infection status is too coarse to reveal an effect, as behavioral or physiological effects of DFTD may not manifest during early infection but increase with tumor burden (Ruiz‐Aravena et al., 2018). Furthermore, only 24 devils in our sample of 90 were infected with DFTD; therefore, our sample size may be too small to reveal any subtle effects or significant interaction terms relating to the effect of DFTD upon isotopic variability. Our dataset includes a number of devils unlikely to display clinical signs of DFTD due to their age; most of the transmission‐relevant injurious biting occurs during the mating season between adults (Hamede et al., 2008; Hamilton et al., 2019). If there is an effect of DFTD upon isotopic signatures, this could influence our findings related to age and isotopic variability due to the age skew of the diseased population. To account for this, we included an interaction between DFTD infection status and age in our GLMs, though this was not retained in any top model.

In conclusion, Tasmanian devil isotopic values and isotopic niche area change with age, likely following a pattern of increased prey size and reduced dietary niche breadth with increasing devil maturity, alongside a reduction in the influence of weaning from a diet of maternal milk. The abundance of devils has declined dramatically due to outbreaks of DFTD, resulting in changing age structures, behaviors, and ecological interactions. Moving forward, this research provides a basis to consider in more detailed terms the potential impacts of DFTD‐related decline upon the trophic ecology of Tasmanian devils and the ecological communities they occupy and influence.

## CONFLICT OF INTEREST

None declared.

## AUTHOR CONTRIBUTIONS

**Olivia Bell:** Conceptualization (lead); data curation (lead); formal analysis (lead); funding acquisition (lead); investigation (lead); methodology (lead); project administration (lead); validation (lead); visualization (lead); writing – original draft (lead); writing – review & editing (lead). **Menna E. Jones:** Conceptualization (equal); data curation (equal); investigation (equal); methodology (equal); resources (equal); supervision (equal); writing – original draft (supporting); writing – review & editing (equal). **Manuel Ruiz‐Aravena:** Data curation (supporting); investigation (supporting); methodology (supporting); writing – review & editing (supporting). **Rodrigo K. Hamede:** Conceptualization (supporting); data curation (supporting); investigation (supporting); methodology (supporting); resources (supporting); supervision (supporting); writing – review & editing (supporting). **Stuart Bearhop:** Conceptualization (equal); formal analysis (equal); methodology (equal); software (equal); supervision (equal); validation (equal); writing – original draft (supporting); writing – review & editing (equal). **Robbie A. McDonald:** Conceptualization (equal); funding acquisition (equal); investigation (equal); methodology (equal); project administration (equal); resources (equal); supervision (equal); writing – original draft (supporting); writing – review & editing (equal).

## Data Availability

Data are available from the Dryad Digital Repository: https://doi.org/10.5061/dryad.m905qftz8 (Bell et al., 2020).

## References

[ece36513-bib-0080] Albanese, S , Dacar, M.A. , & Ojeda, R.A. (2012). Unvarying diet of a Neotropical desert marsupial inhabiting a variable environment: the case of *Thylamys pallidior* . Acta Theriologica, 57, 185–188.

[ece36513-bib-0001] Andersen, G. E. , Johnson, C. N. , Barmuta, L. A. , & Jones, M. E. (2017). Dietary partitioning of Australia's two marsupial hypercarnivores, the Tasmanian devil and the spotted‐tailed quoll, across their shared distributional range. PLoS One, 12, e0188529 10.1371/journal.pone.0188529 29176811 PMC5703475

[ece36513-bib-0002] Anderson, O. R. J. , Phillips, R. A. , Shore, R. F. , McGill, R. A. R. , McDonald, R. A. , & Bearhop, S. (2009). Diet, individual specialisation and breeding of brown skuas (*Catharacta antarctica lonnbergi*): An investigation using stable isotopes. Polar Biology, 32, 27–33. 10.1007/s00300-008-0498-9

[ece36513-bib-0003] Attard, M. R. G. , Chamoli, U. , Ferrara, T. L. , Rogers, T. L. , & Wroe, S. (2011). Skull mechanics and implications for feeding behaviour in a large marsupial carnivore guild: The thylacine, Tasmanian devil and spotted‐tailed quoll. Journal of Zoology, 285, 292–300. 10.1111/j.1469-7998.2011.00844.x

[ece36513-bib-0078] Bartoń, K. (2018). MuMin: Multi‐model inference. http://r‐forge.r‐project.org/projects/mumin/

[ece36513-bib-0004] Bearhop, S. , Adams, C. E. , Waldron, S. , Fuller, R. A. , & Macleod, H. (2004). Determining trophic niche width: A novel approach using stable isotope analysis. Journal of Animal Ecology, 73, 1007–1012. 10.1111/j.0021-8790.2004.00861.x

[ece36513-bib-0005] Bearhop, S. , Waldron, S. , Votier, S. C. , & Furness, R. W. (2002). Factors that influence assimilation rates and fractionation of nitrogen and carbon stable isotopes in avian blood and feathers. Physiological and Biochemical Zoology, 75, 451–458. 10.1086/342800 12529846

[ece36513-bib-0006] Bell, O. , Jones, M. E. , Ruiz‐Aravena, M. , Hamede, R. K. , Bearhop, S. , & McDonald, R. A. (2020). Data from: Age‐related variation in the trophic characteristics of a marsupial carnivore, the Tasmanian devil *Sarcophilus harrisii* . Dryad Digital Repository, 10.5061/dryad.m905qftz8 PMC7391331 32760570

[ece36513-bib-0007] Bennett, A. , & Baxter, B. (1989). Diet of the long‐nosed potoroo, *Potorous tridactylus* (Marsupialia, Potoroidae), in Southwestern Victoria. Wildlife Research, 16, 263 10.1071/WR9890263

[ece36513-bib-0008] Bodey, T. W. , Bearhop, S. , Roy, S. S. , Newton, J. , & McDonald, R. A. (2010). Behavioural responses of invasive American mink *Neovison vison* to an eradication campaign, revealed by stable isotope analysis. Journal of Applied Ecology, 47, 114–120. 10.1111/j.1365-2664.2009.01739.x

[ece36513-bib-0009] Bodey, T. W. , Cleasby, I. R. , Votier, S. C. , Hamer, K. C. , Newton, J. , Patrick, S. C. , … Bearhop, S. (2018). Frequency and consequences of individual dietary specialisation in a wide‐ranging marine predator, the northern gannet. Marine Ecology Progress Series, 604, 251–262. 10.3354/meps12729

[ece36513-bib-0010] Bolnick, D. I. , Svanbäck, R. , Fordyce, J. A. , Yang, L. H. , Davis, J. M. , Hulsey, C. D. , & Forister, M. L. (2003). The ecology of individuals: Incidence and implications of individual specialization. American Naturalist, 161, 1–28. 10.1086/343878 12650459

[ece36513-bib-0011] Crawford, K. , McDonald, R. A. , & Bearhop, S. (2008). Applications of stable isotope techniques to the ecology of mammals. Mammal Review, 38, 87–107. 10.1111/j.1365-2907.2008.00120.x

[ece36513-bib-0012] Cummings, D. O. , Buhl, J. , Lee, R. W. , Simpson, S. J. , & Holmes, S. P. (2012). Estimating niche width using stable isotopes in the face of habitat variability: A modelling case study in the marine environment. PLoS One, 7, 40539 10.1371/journal.pone.0040539 PMC3410910 22876280

[ece36513-bib-0013] Cunningham, C. X. , Johnson, C. N. , Barmuta, L. A. , Hollings, T. , Woehler, E. J. , & Jones, M. E. (2018). Top carnivore decline has cascading effects on scavengers and carrion persistence. Proceedings of the Royal Society B‐Biological Sciences, 285(1892), 20181582 10.1098/rspb.2018.1582 PMC6283947 30487308

[ece36513-bib-0014] Cunningham, C. X. , Johnson, C. N. , Hollings, T. , Kreger, K. , & Jones, M. E. (2019). Trophic rewilding establishes a landscape of fear: Tasmanian devil introduction increases risk‐sensitive foraging in a key prey species. Ecography, 42, 1–7. 10.1111/ecog.04635

[ece36513-bib-0015] DeNiro, M. J. , & Epstein, S. (1978). Influence of diet on the distribution of carbon isotopes in animals. Geochimica et Cosmochimica Acta, 42, 495–506. 10.1016/0016-7037(78)90199-0

[ece36513-bib-0016] Dickman, C. R. (1988). Age‐related dietary change in the European hedgehog, *Erinaceus europaeus* . Journal of Zoology, 215, 1–14. 10.1111/j.1469-7998.1988.tb04881.x

[ece36513-bib-0017] Fahy, G. E. , Richards, M. P. , Fuller, B. T. , Deschner, T. , Hublin, J. J. , & Boesch, C. (2014). Stable nitrogen isotope analysis of dentine serial sections elucidate sex differences in weaning patterns of wild chimpanzees (*Pan troglodytes*). American Journal of Physical Anthropology, 153, 635–642. 10.1002/ajpa.22464 24395019

[ece36513-bib-0079] Evacitas, F.C. , Kao, W.Y. , Worthy, G.A. , & Chao, L.S. (2017). Annual variability in dentin δ15N and δ13C reveal sex differences in weaning age and feeding habits in Risso's dolphins (*Grampus griseus*). Marine Mammal Science, 33, 748–770.

[ece36513-bib-0018] Fisher, P. (1999). Review of using rhodamine B as a marker for wildlife studies. Wildlife Society Bulletin, 27, 318–329. 10.1098/rstb.2010.0081

[ece36513-bib-0019] Geipel, I. , Kalko, E. K. V. , Wallmeyer, K. , & Knörnschild, M. (2013). Postweaning maternal food provisioning in a bat with a complex hunting strategy. Animal Behavior, 85, 1435–1441. 10.1016/j.anbehav.2013.03.040

[ece36513-bib-0020] Guiler, E. R. (1970). Observations on the Tasmanian devil, *Sarcophilus harrisii* (Marsupialia: Dasyuridae) II. Reproduction, breeding, and growth of pouch young. Australian Journal of Zoology, 18, 63–70.

[ece36513-bib-0021] Hamede, R. K. , McCallum, H. , & Jones, M. (2008). Seasonal, demographic and density‐related patterns of contact between Tasmanian devils (*Sarcophilus harrisii*): Implications for transmission of devil facial tumour disease. Austral Ecology, 33, 614–622. 10.1111/j.1442-9993.2007.01827.x

[ece36513-bib-0022] Hamilton, D. G. , Jones, M. E. , Cameron, E. Z. , McCallum, H. , Storfer, A. , Hohenlohe, P. A. , & Hamede, R. K. (2019). Rate of intersexual interactions affects injury likelihood in Tasmanian devil contact networks. Behavioral Ecology, 30, 1087–1095. 10.1093/beheco/arz054

[ece36513-bib-0023] Hammerschlag‐Peyer, C. M. , Yeager, L. A. , Araújo, M. S. , & Layman, C. A. (2011). A hypothesis‐testing framework for studies investigating ontogenetic niche shifts using stable isotope ratios. PLoS One, 6, e27104 10.1371/journal.pone.0027104 22073265 PMC3207812

[ece36513-bib-0024] Hawkins, C. E. , Baars, C. , Hesterman, H. , Hocking, G. J. , Jones, M. E. , Lazenby, B. , … Wiersma, J. (2006). Emerging disease and population decline of an island endemic, the Tasmanian devil *Sarcophilus harrisii* . Biological Conservation, 131, 307–324. 10.1016/j.biocon.2006.04.010

[ece36513-bib-0025] Hesterman, H. , Jones, S. M. , & Schwarzenberger, F. (2008). Reproductive endocrinology of the largest dasyurids: Characterization of ovarian cycles by plasma and fecal steroid monitoring. Part I. The Tasmanian devil (*Sarcophilus harrisii*). General and Comparative Endocrinology, 155, 234–244. 10.1016/j.ygcen.2007.05.013 17592734

[ece36513-bib-0026] Hobson, K. A. , & Clark, R. G. (1992). Assessing avian diets using stable isotopes II: Factors influencing diet‐tissue fractionation. Condor, 94, 189–197. 10.2307/1368808

[ece36513-bib-0082] Hobson, K.A. , & Sease, J.L. (1998). Stable isotope analyses of tooth annuli reveal temporal dietary records: an example using Steller sea lions. Marine Mammal Science, 14, 116–129.

[ece36513-bib-0027] Hollings, T. , Jones, M. , Mooney, N. , & McCallum, H. (2014). Trophic cascades following the disease‐induced decline of an apex predator, the Tasmanian devil. Conservation Biology, 28, 63–75. 10.1111/cobi.12152 24024987

[ece36513-bib-0028] Hollings, T. , McCallum, H. , Kreger, K. , Mooney, N. , & Jones, M. (2015). Relaxation of risk‐sensitive behaviour of prey following disease‐induced decline of an apex predator, the Tasmanian devil. Proceedings of the Royal Society B: Biological Sciences, 282(1810), 20150124 10.1098/rspb.2015.0124 PMC4590467 26085584

[ece36513-bib-0029] Inger, R. , Ruxton, G. D. , Newton, J. , Colhoun, K. , Robinson, J. A. , Jackson, A. L. , & Bearhop, S. (2006). Temporal and intrapopulation variation in prey choice of wintering geese determined by stable isotope analysis. Journal of Animal Ecology, 75, 1190–1200. 10.1111/j.1365-2656.2006.01142.x 16922855

[ece36513-bib-0030] Jackson, A. L. , Inger, R. , Parnell, A. C. , & Bearhop, S. (2011). Comparing isotopic niche widths among and within communities: SIBER ‐ Stable Isotope Bayesian Ellipses in R. Journal of Animal Ecology, 80, 595–602. 10.1111/j.1365-2656.2011.01806.x 21401589

[ece36513-bib-0031] Johnson, C. K. , Tinker, M. T. , Estes, J. A. , Conrad, P. A. , Staedler, M. , Miller, M. A. , … Mazet, J. A. K. (2009). Prey choice and habitat use drive sea otter pathogen exposure in a resource‐limited coastal system. Proceedings of the National Academy of Sciences of the Unites States of America, 106, 2242–2247. 10.1073/pnas.0806449106 PMC2650139 19164513

[ece36513-bib-0032] Jones, M. E. (1995). Guild structure of the large marsupial carnivores in Tasmania. University of Tasmania. PhD Thesis.

[ece36513-bib-0033] Jones, M. (1997). Character displacement in Australian dasyurid carnivores: Size relationships and prey size patterns. Ecology, 78, 2569–2587. 10.1890/0012-9658(1997)078[2569:CDIADC]2.0.CO;2

[ece36513-bib-0034] Jones, M. E. , & Barmuta, L. A. (1998). Diet overlap and relative abundance of sympatric dasyurid carnivores: A hypothesis of competition. Journal of Animal Ecology, 67, 410–421. 10.1046/j.1365-2656.1998.00203.x

[ece36513-bib-0035] Jones, M. E. , & Barmuta, L. A. (2000). Niche differentiation among sympatric Australian Dasyurid carnivores. Journal of Mammalogy, 81, 434–447. 10.1644/1545-1542(2000)081<0434

[ece36513-bib-0036] Jones, M. E. , Cockburn, A. , Hamede, R. , Hawkins, C. , Hesterman, H. , Lachish, S. , … Pemberton, D. (2008). Life‐history change in disease‐ravaged Tasmanian devil populations. Proceedings of the National Academy of Sciences, 105, 10023–10027. 10.1073/pnas.0711236105 PMC2481324 18626026

[ece36513-bib-0037] Keeley, T. , Russell, T. , Carmody, K. , Kirk, G. , Eastley, T. , Britt‐Lewis, A. , … Hughes, R. L. (2017). Seasonality and breeding success of captive and wild Tasmanian devils (*Sarcophilus harrisii*). Theriogenology, 95, 33–41. 10.1016/j.theriogenology.2017.02.013 28460677

[ece36513-bib-0038] Knoff, A. , Hohn, A. , & Macko, S. (2008). Ontogenetic diet changes in bottlenose dolphins (*Tursiops truncatus*) reflected through stable isotopes. Marine Mammal Science, 24, 128–137. 10.1111/j.1748-7692.2007.00174.x

[ece36513-bib-0039] Lachish, S. , Jones, M. , & McCallum, H. (2007). The impact of disease on the survival and population growth rate of the Tasmanian devil. Journal of Animal Ecology, 76, 926–936. 10.1111/j.1365-2656.2007.01272.x 17714271

[ece36513-bib-0040] Lachish, S. , McCallum, H. , & Jones, M. (2009). Demography, disease and the devil: Life‐history changes in a disease‐affected population of Tasmanian devils (*Sarcophilus harrisii*). Journal of Animal Ecology, 78, 427–436. 10.1111/j.1365-2656.2008.01494.x 19021786

[ece36513-bib-0041] Lachish, S. , Miller, K. J. , Storfer, A. , Goldizen, A. W. , & Jones, M. E. (2011). Evidence that disease‐induced population decline changes genetic structure and alters dispersal patterns in the Tasmanian devil. Heredity, 106, 172–182. 10.1038/hdy.2010.17 20216571 PMC3183847

[ece36513-bib-0042] Layman, C. A. , Araujo, M. S. , Boucek, R. , Hammerschlag‐Peyer, C. M. , Harrison, E. , Jud, Z. R. , … Bearhop, S. (2012). Applying stable isotopes to examine food‐web structure: An overview of analytical tools. Biological Reviews, 87, 545–562. 10.1111/j.1469-185X.2011.00208.x 22051097

[ece36513-bib-0043] Lee, P. C. , Majluf, P. , & Gordon, I. J. (1991). Growth, weaning and maternal investment from a comparative perspective. Journal of Zoology, 225, 99–114. 10.1111/j.1469-7998.1991.tb03804.x

[ece36513-bib-0044] Loh, R. , Bergfeld, J. , Hayes, D. , O'Hara, A. , Pyecroft, S. , Raidal, S. , & Sharpe, R. (2006). The pathology of devil facial tumor disease (DFTD) in Tasmanian Devils (*Sarcophilus harrisii*). Veterinary Pathology, 43, 890–895. 10.1354/vp.43-6-890 17099145

[ece36513-bib-0081] Martins E.G. , Araújo M.S. , Bonato V. , dos Reis S.F. (2008). Sex and season affect individual‐level diet variation in the Neotropical marsupial *Gracilinanus microtarsus* (Didelphidae). Biotropica, 40, 132–135.

[ece36513-bib-0045] McCallum, H. , Jones, M. , Hawkins, C. , Hamede, R. , Lachish, S. , Sinn, D. L. , … Lazenby, B. (2009). Transmission dynamics of Tasmanian devil facial tumor disease may lead to disease‐induced extinction. Ecology, 90, 3379–3392. 10.1890/08-1763.1 20120807

[ece36513-bib-0046] Newsome, S. D. , del Martinez del Rio, C. , Bearhop, S. , & Phillips, D. L. (2007). A niche for isotope ecology. Frontiers in Ecology and the Environment, 5, 429–436. 10.1890/060150.01

[ece36513-bib-0047] Newsome, S. D. , Etnier, M. A. , Monson, D. H. , & Fogel, M. L. (2009). Retrospective characterization of ontogenetic shifts in killer whale diets via δ13C and δ15N analysis of teeth. Marine Ecology Progress Series, 374, 229–242. 10.3354/meps07747

[ece36513-bib-0048] Newsome, S. D. , Tinker, M. T. , Monson, D. H. , Oftedal, O. T. , Ralls, K. , Staedler, M. M. , … Estes, J. A. (2009). Using stable isotopes to investigate individual diet specialization in California sea otters (*Enhydra lutris nereis*). Ecology, 90, 961–974. 10.1890/07-1812.1 19449691

[ece36513-bib-0049] Olson, M. H. (1996). Ontogenetic niche shifts in largemouth bass: Variability and consequences for first‐year growth. Ecology, 77, 179–180. 10.2307/2265667

[ece36513-bib-0050] Orr, A. J. , Newsome, S. D. , Laake, J. L. , Vanblaricom, G. R. , & Delong, R. L. (2012). Ontogenetic dietary information of the California sea lion (*Zalophus californianus*) assessed using stable isotope analysis. Marine Mammal Science, 28, 714–732. 10.1111/j.1748-7692.2011.00522.x

[ece36513-bib-0051] Patrick, S. C. , Bearhop, S. , Bodey, T. W. , Grecian, W. J. , Hamer, K. C. , Lee, J. , & Votier, S. C. (2015). Individual seabirds show consistent foraging strategies in response to predictable fisheries discards. Journal of Avian Biology, 46, 431–440. 10.1111/jav.00660

[ece36513-bib-0052] Pearse, A. M. , & Swift, K. (2006). Transmission of devil facial‐tumour disease. Nature, 439, 549 10.1038/439549a 16452970

[ece36513-bib-0053] Pemberton, D. (1990). Social organisation and behaviour of the Tasmanian devil, Sarcophilus harrisii. Hobart: University of Tasmania.

[ece36513-bib-0054] Pemberton, D. , & Renouf, D. (1993). A field study of communication and social behaviour of the Tasmanian devil at feeding sites. Australian Journal of Zoology, 41, 507–526. 10.1071/ZO9930507

[ece36513-bib-0055] Pharo, E. A. (2019). Marsupial milk: A fluid source of nutrition and immune factors for the developing pouch young. Reproduction, Fertility, and Development, 31, 1252–1265. 10.1071/RD18197 30641029

[ece36513-bib-0056] Phillips, R. A. , McGill, R. A. R. , Dawson, D. A. , & Bearhop, S. (2011). Sexual segregation in distribution, diet and trophic level of seabirds: Insights from stable isotope analysis. Marine Biology, 158, 2199–2208. 10.1007/s00227-011-1725-4

[ece36513-bib-0057] Polis, G. A. (1984). Age structure component of niche width and intraspecific resource partitioning: Can age groups function as ecological species? American Naturalist, 123, 541–564. 10.1086/284221

[ece36513-bib-0058] Pye, R. , Hamede, R. , Siddle, H. V. , Caldwell, A. , Knowles, G. W. , Swift, K. , … Woods, G. M. (2016). Demonstration of immune responses against devil facial tumour disease in wild Tasmanian devils. Biology Letters, 12, 20160553 10.1098/rsbl.2016.0553 28120799 PMC5095191

[ece36513-bib-0059] Pyecroft, S. B. , Pearse, A. M. , Loh, R. , Swift, K. , Belov, K. , Fox, N. , … Moore, R. (2007). Towards a case definition for devil facial tumour disease: What is it? EcoHealth, 4, 346–351. 10.1007/s10393-007-0126-0

[ece36513-bib-0060] Quin, D. G. (1988). Observations on the diet of the southern brown bandicoot, *Isoodon obesulus* (Marsupialia: Peramelidae) in Southern Tasmania. Australian Mammalogy, 11, 15–25.

[ece36513-bib-0061] R Core Team (2018). R: A language and environment for statistical computing. Vienna, Austria: R Foundation for Statistical Computing Retrieved from http://www.r‐project.org/index.html

[ece36513-bib-0062] Reich, K. J. , Bjorndal, K. A. , & Bolten, A. B. (2007). The ‘lost years’ of green turtles: Using stable isotopes to study cryptic lifestages. Biology Letters, 3, 712–714. 10.1098/rsbl.2007.0394 17878144 PMC2391226

[ece36513-bib-0063] Riccialdelli, L. , Newsome, S. D. , Dellabianca, N. A. , Bastida, R. , Fogel, M. L. , & Goodall, R. N. P. (2013). Ontogenetic diet shift in Commerson's dolphin (*Cephalorhynchus commersonii commersonii*) off Tierra del Fuego. Polar Biology, 36, 617–627. 10.1007/s00300-013-1289-5

[ece36513-bib-0064] Ridpath, M. G. , & Moreau, R. E. (1965). The birds of Tasmania: Ecology and distributions. Ibis, 108, 348–393.

[ece36513-bib-0065] Robertson, A. , McDonald, R. A. , Delahay, R. J. , Kelly, S. D. , & Bearhop, S. (2014). Individual foraging specialisation in a social mammal: The European badger (*Meles meles*). Oecologia, 176, 409–421. 10.1007/s00442-014-3019-2 25037464

[ece36513-bib-0067] Ruiz‐Aravena, M. , Jones, M. E. , Carver, S. , Estay, S. , Espejo, C. , Storfer, A. , & Hamede, R. K. (2018). Sex bias in ability to cope with cancer: Tasmanian devils and facial tumour disease. Proceedings of the Royal Society B‐Biological Sciences, 285(1891), 20182239 10.1098/rspb.2018.2239 PMC6253378 30464069

[ece36513-bib-0068] Sheppard, C. E. , Inger, R. , McDonald, R. A. , Barker, S. , Jackson, A. L. , Thompson, F. J. , … Marshall, H. H. (2018). Intragroup competition predicts individual foraging specialisation in a group‐living mammal. Ecology Letters, 21, 665–673. 29542220 10.1111/ele.12933 PMC5947261

[ece36513-bib-0069] Stauss, C. , Bearhop, S. , Bodey, T. W. , Garthe, S. , Gunn, C. , Grecian, W. J. , … Votier, S. C. (2012). Sex‐specific foraging behaviour in northern gannets *Morus bassanus*: Incidence and implications. Marine Ecology Progress Series, 457, 151–162. 10.3354/meps09734

[ece36513-bib-0083] Strahan, R. (1983). Complete book of Australian mammals, Sydney: Angus & Robertson.

[ece36513-bib-0070] Swan, G. J. F. , Redpath, S. M. , Bearhop, S. , & McDonald, R. A. (2017). Ecology of problem individuals and the efficacy of selective wildlife management. Trends in Ecology & Evolution, 32, 518–530. 10.1016/j.tree.2017.03.011 28529028

[ece36513-bib-0071] Tanner, J. B. , Zelditch, M. L. , Lundrigan, B. L. , & Holekamp, K. E. (2010). Ontogenetic change in skull morphology and mechanical advantage in the spotted hyena (*Crocuta crocuta*). Journal of Morphology, 271, 353–365. 10.1002/jmor.10802 19862838

[ece36513-bib-0072] Taylor, R. J. (2006). Seasonal changes in the diet of the Tasmanian Bettong (*Bettongia gaimardi*), a mycophagous marsupial. Journal of Mammalogy, 73, 408–414. 10.2307/1382076

[ece36513-bib-0073] Tieszen, L. L. , Boutton, T. W. , Tesdahl, K. G. , & Slade, N. A. (1983). Fractionation and turnover of stable carbon isotopes in animal tissues: Implications for δ13C analysis of diet. Oecologia, 57, 32–37. 10.1007/BF00379558 28310153

[ece36513-bib-0074] Vales, D. G. , Cardona, L. , García, N. A. , Zenteno, L. , & Crespo, E. A. (2015). Ontogenetic dietary changes in male South American fur seals *Arctocephalus australis* in Patagonia. Marine Ecology Progress Series, 525, 245–260. 10.3354/meps11214

[ece36513-bib-0075] Votier, S. C. , Bearhop, S. , Witt, M. J. , Inger, R. , Thompson, D. , & Newton, J. (2010). Individual responses of seabirds to commercial fisheries revealed using GPS tracking, stable isotopes and vessel monitoring systems. Journal of Applied Ecology, 47, 487–497. 10.1111/j.1365-2664.2010.01790.x

[ece36513-bib-0076] Werner, E. E. , & Gilliam, J. F. (1984). The ontogenetic niche and species interactions in size‐structured populations. Annual Review of Ecology and Systematics, 15, 393–425. 10.1146/annurev.es.15.110184.002141

[ece36513-bib-0077] Wroe, S. , McHenry, C. , & Thomason, J. (2005). Bite club: Comparative bite force in big biting mammals and the prediction of predatory behaviour in fossil taxa. Proceedings of the Royal Society B‐Biological Sciences, 272, 619–625. 10.1098/rspb.2004.2986 PMC1564077 15817436

